# Twig biomass allocation of *Betula platyphylla* in different habitats in Wudalianchi Volcano, northeast China

**DOI:** 10.1515/biol-2021-0078

**Published:** 2021-08-09

**Authors:** Fan Yang, Lihong Xie, Qingyang Huang, Hongjie Cao, Jifeng Wang, Jianbo Wang, Yingnan Liu, Hongwei Ni, Liqiang Mu

**Affiliations:** Forest Plant of Resources, College of Forestry, Northeast Forestry University, No. 26, Hexing Road, Xiangfang District, Harbin, 150040, China; Ecological Environment Center, Institute of Natural Resources and Ecology, Heilongjiang Academy of Sciences/National and Provincial Joint Engineering Laboratory of Wetlands and Ecological Conservation, No. 103, Haping Road, Xiangfang District, Harbin, 150040, China; College of Forestry, Heilongjiang Academy of Forestry, No. 134, Haping Road, Xiangfang District, Harbin, 150040, China

**Keywords:** twig traits, *Betula platyphylla*, biomass allocation, volcano

## Abstract

Understanding the response of biomass allocation in current-year twigs is crucial for elucidating the plant life-history strategies under heterogeneous volcanic habitats. We aimed to test whether twig biomass allocation, within-leaf biomass allocation, and the size-number trade-off of *Betula platyphylla* would be influenced. We measured twig traits of *B. platyphylla* in Wudalianchi volcanic kipuka, the lava platform, and Shankou lake in northeastern China using standardized major axis analyses. The results showed that the leaf number, total lamina mass (TLAM), stem mass (SM), and twig mass (TM) were significantly different between the three habitats and were greatest in kipuka with abundant soil nutrients. TLAM and SM scaled allometrically with respect to TM, while the normalization constants of the lava platform differ significantly between kipuka and Shankou lake, which showed that under certain TM, leaves gain more biomass in the lava platform. However, within the leaf, individual lamina mass (ILM) scaled isometrically with respect to individual petiole mass (IPM) in kipuka and the lava platform, but ILM scaled allometrically to IPM in Shankou lake. Our results indicated that inhabitats influenced the twig traits and biomass allocation and within-leaf biomass allocation are strategies for plants to adapt to volcanic heterogeneous habitats.

## 1 Introduction

Twig biomass allocation is an important driving factor for capturing the net carbon affecting the phenotype and function of plant leaves and twigs and is sensitive to environmental change. Research on the effects of heterogeneous habitats on biomass allocation is crucial for understanding the plant life-history strategies [1,2]. The allometric function has been applied to describe plant biomass allocation. The allometry estimates how one variable scales against another and tests hypotheses about the nature of this relationship and how it varies across samples [3,28]. The current-year twig is the most viable part of the plant branching system, its internal nutrient transformation rate is high, and its trait response is easily observed. The current-year twig can reflect the response of plants to the environment more accurately than the older parts of the plant. Therefore, revealing the relationships of the internal components of laminas, petioles, and stems on the current-year twig is vital for understanding the resource allocation strategies of plants under environmental stress [4].

Plants are sessile and grow in a specific environment. Because the available resources are limited, plants use resources more efficiently by adjusting traits. Plants invest too many resources in a particular functional trait, and the corresponding traits will be reduced [5,6]. The trade-off relationship between twig and leaf size is the core phenomenon in the study of plant life-history strategies. Twigs and leaves are important transport and material production organs that play a critical role in carbon acquisition, allocation strategy, and water transport efficiency [4,7]. Some studies have suggested that the leaf mass scales isometrically with the stem mass (SM) in twigs, and the twig size does not significantly affect the allocation pattern [8]. In determining the relationship between twigs and leaves, the thicker the twigs, the larger the components (leaves, inflorescences, and fruits), and larger leaf biomass [7,9,10]. The relationship also exists within the leaf. The leaf is composed of a lamina and petiole that is an essential component of the twig. The lamina is the primary site of photosynthetic activity and carbohydrate synthesis, while the petiole is a cantilevered structure that supports and supplies laminas, additionally playing the role of supporting static gravity and resisting external dynamic tension [11]. Generally speaking, when the biomass of the total leaves is fixed, the more biomass allocated to leaves, the stronger the photosynthetic carbon acquisition capacity of leaves, and the more favorable to plants; however, the increase of leaf area and weight requires that the petiole must have higher support capacity, and the increase of leaves also leads to an increase of biomass allocated to petioles and midvein [12]. Numerous studies of twig biomass allocation have been carried out on different habitats [13] and plant species [14,15]; however, there are a few studies on volcanic habitats. Therefore, further research on how plants adjust the relationship between twigs and leaves to adapt to extreme volcanic habitats is required.

Besides the trade-off relationship of biomass allocation, the trade-off between the leaf size and leaf number (LN) has a very important influence on the plant biomass allocation strategies; it affects the plasticity of the leaf economic spectrum and leaf function traits [16] and reflects the plant’s adaptability to special habitats [17]. Westby found a trade-off relationship between the leaf size and LN at the twig level [18]. Kleiman and Aarssen studied 24 deciduous broadleaved tree species and found a negative isokinetic growth relationship between leafing intensity (LI) and leaf area [18]. Li et al. study on 12 deciduous shrub species in the western Gobi and 56 woody plants in temperate zones also showed an isokinetic growth relationship between the leaf size and LI [19]. However, Milla found a negative allometric growth relationship between the dry leaf weight and LI [20]. Although the trade-off between the leaf size and LI is widespread among species and habitats [21,22], a few research studies have focused on the relationship between different volcanic habitats.

The Wudalianchi National Geological Park (WNGK), located in the Heilongjiang Province of Northeast China, has a well-preserved single genetic inland volcanic landform and maintains the original and complete vegetation ecological succession process. Thus, it provides an ideal location for the study of plant succession and evolution in a volcanic ecosystem. After the volcanic eruption, two types of relative volcanic landforms were created, the lava platform and the kipuka. Although in the same area, Shankou lake was not affected by volcanic activity. *Betula platyphylla* is a tall deciduous tree, a pioneer species distributed in a kipuka, lava platform, and Shankou lake. These three habitats exhibit differences in environmental factors such as light, water, and soil nutrients. In this study, twig traits and biomass allocation, and the size-number trade-offs of *B. platyphylla* in three different habitats were investigated. The objective was to understand the adaptive strategies adopted by adjusting twig biomass allocation of *B. platyphylla* to provide a scientific basis for the study of plant succession in a volcanic habitat.

## 2 Materials and methods

### 2.1 Study area

This study was conducted in WNGK, Heilongjiang Province, northeast China (48°30′–48°50′N, 126°00′–126°25′E). The last volcanic eruption occurred in Laoheishan between 1719 and 1721, forming an 80 km^2^ basalt lava platform [23]. The kipuka was not covered with volcanic lava and retained the original island soil [24]. Shankou Park, 65 km from WNGK, was not affected by the volcanic eruption [25] (Table 1). The Wudalianchi area has a temperate continental monsoon climate with severely cold and long winters and pleasantly cool and short summers. It has an annual average temperature of −0.5°C, annual average precipitation of 473 mm, an annual frost-free period of 121 days, and a zonal dark-brown soil type. The vegetation of Wudalianchi is temperate, broad-leaf, and mixed forest. The pioneer community of forest succession in the three habitats was Populus birch forest. The dominant plants are *Larix gmelini*, *Quercus mongolica*, *B. platyphylla*, *Populus davidiana*, and *P. koreana*.

**Table 1:** General characteristics of the study areas

Plots	Kipuka (H1)	Lava platform (H2)	Shankou Park (H3)
Location	48°39ʹ13ʺN, 126°16ʹ30ʺE	48°42ʹ32ʺN, 126°07ʹ06ʺE	48°28ʹ20ʺN, 126°30ʹ30ʺE
Eruption time	300 years ago	300 years ago	No eruption
Soil type	Dark-brown soil, black volcanic ash [26]	Volcanic stony soil, Herbaceous volcanic ash [26]	Dark-brown soil
TN (%)	1.72 ± 0.35	0.17 ± 0.07	0.58 ± 0.2
TP (%)	0.17 ± 0.03	0.31 ± 0.05	0.14 ± 0.05
TK (%)	1.51 ± 0.31	3.24 ± 0.31	1.76 ± 0.18
Altitude	328 m	328 m	306 m
Vegetation	Poplar birch forest	Poplar birch forest	Poplar birch forest
Crown density (%)	60–80	30–50	60–80
Height (m)	7–11	3–5.5	10–16
Diameter at breast height (cm)	6.8–8.5	2.6–6.8	12–13.5
Foreat age	28 ± 6.7	45 ± 8.2	43 ± 2.4

In August 2019, five healthy, mature *B. platyphylla* plants were selected from the kipuka, lava platform, and Shankou lake. To reduce the influence of the tree size, the age of sample trees was not less than 20 years, and they had similar diameters at breast height. The distance between samples was not less than 20 m. The top six vegetative branches without apparent leaf area loss were randomly selected from the outer canopy of each *B. platyphylla* plant, that is, from the distal end to the last terminal twig that is usually branchless, flowerless, and fruitless; it is the terminal twig of the current year’s twig.

Twigs are composed of leaves and stems, and the leaves consist of two parts: lamina and petiole. All leaves on each current year’s twig were taken off, and the LN lamina was recorded. The laminas, petioles, and stems were oven-dried at 70°C for 48 h to a constant weight, and the dry weight was measured. In this study, the total lamina mass (TLAM), total petiole mass (TPM), and total leaf mass (TLM) were determined using the total dry weights of the laminas, petioles, and stems of each twig.

Within the leaf biomass and trade-off relationship, individual leaf mass is the average of all the individual leaf dry weights. Additionally, we calculated the leaf intensity using the relevant calculation formula as follows:

\begin{array}{c}\text{Leaf}\hspace{.25em}\text{intensity}\hspace{.25em}(\text{No}\text{.}/\text{g})=\text{leaf}\hspace{.5em}\text{number}\hspace{.25em}\text{per}\hspace{.25em}\text{unit}\hspace{.25em}\\ \text{twig/twig}\hspace{.25em}\text{mass}\hspace{.25em}(\text{including}\hspace{.25em}\text{stem,}\hspace{.25em}\text{laminas,}\hspace{.25em}\text{and}\hspace{.25em}\text{petioles}).\end{array}]

### 2.2 Statistical analysis

All the data were log 10 transformed to fit a normal distribution before analysis. The mean and standard error of each twig trait was calculated. Analysis of variance followed by Duncan’s test was used to identify significant differences among the habitats. The relationships between TLAM and twig mass (TM), TPM and TM, SM and TM, TLM and SM, ILM and individual petiole mass (IPM), and ILM and LI were evaluated by regression analysis. Regression analyses showed that the variables of primary interest were log–log linear-correlated and conformed to the equation log(*y*) = log(*b*) + *a* log(*x*), where log *b* is the scaling constant, *a* is the scaling exponent, and *y* and *x* are different parts of plant biomass. When *a* = 1, the scaling relationship is isometric; and when *a* ≠ 1, the scaling relationship is allometric [27]. Then, we compared the slopes of these linear relationships during different habitats using standardized major axis regression analysis, which was implemented in the “smatr” package [28]. All statistical analyses were performed using R-3.6.2.

## 3 Results

### 3.1 Twig traits of *B. platyphylla* in heterogeneous habitat

The LN, TLAM, SM, and TM of *B. platyphylla* were significantly different among the three habitats (Table 2). The LN, TLAM, SM, and TM of *B. platyphylla* in the kipuka were greater than those in the lava platform and Shankou lake. The IPM and TPM of *B. platyphylla* in the kipuka were significantly higher than the lava platform and Shankou lake. The LN, TLAM, SM, and TM of *B. platyphylla* were larger in the kipuka but were smaller in the lava platform.

**Table 2:** Branch traits of *B. platyphylla* in a heterogeneous habitat (mean ± SD)

Trait	Codes	Kipuka (H1)	Lava platform (H2)	Shankou lake (H3)
LN	(LN)	8.57 ± 2.21a	4.90 ± 0.80c	6.90 ± 1.49b
ILM (mg)	(ILM)	130.40 ± 42.48a	119.02 ± 24.35a	125.62 ± 57.30a
IPM (mg)	(IPM)	9.20 ± 2.70a	7.72 ± 1.76b	7.42 ± 3.11b
TLAM (g)	(TLAM)	1.17 ± 0.63a	0.59 ± 0.17c	0.87 ± 0.42b
TPM (g)	(TPM)	0.08 ± 0.04a	0.04 ± 0.01b	0.05 ± 0.02b
SM (g)	(SM)	0.30 ± 0.19a	0.08 ± 0.02c	0.21 ± 0.11b
TM (g)	(TM)	1.56 ± 0.85a	0.70 ± 0.20c	1.14 ± 0.54b
LI (No./g)	(LI)	6.46 ± 2.49a	7.31 ± 1.64a	7.11 ± 2.74a

Note: Different lowercase letters mean significant difference at the *α* = 0.05 level between different habitats.

### 3.2 Twig biomass allocation of *B. platyphylla* in a heterogeneous habitat

**Figure 1:**
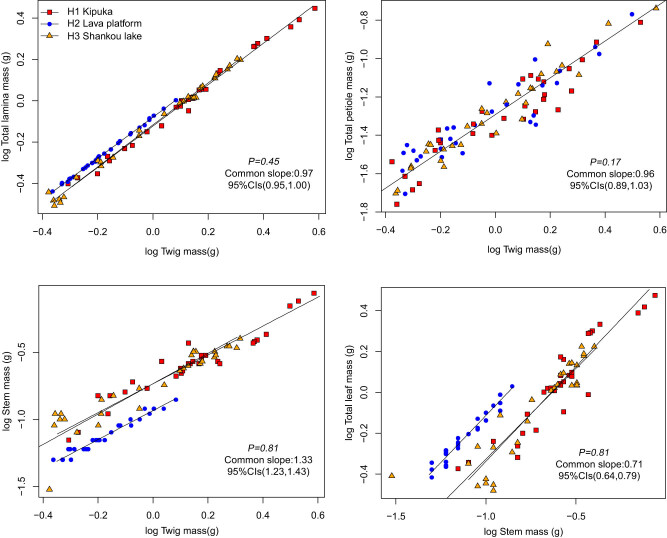
Twig biomass allocation relationship of *B. platyphylla*.

The TM was positively correlated with the TLAM and SM (Figure 1, Table 3). Among the three habitats, TLAM and SM scaled allometrically with respect to TM, with common slopes of 0.97 (95% CI = 0.95 and 1.00, *P* = 0.45) and 1.33 (95% CI = 1.23 and 1.43, *P* = 0.81), and the normalization constants of *B. platyphylla* in lava platform differ significantly in kipuka and Shankou lake. The TM was positively correlated with TPM. Their common slope was 0.96 (95% CI = 0.89 and 1.03, *P* = 0.17). TPM scaled isometrically with respect to TM. The TLM was positively correlated with the SM. Among the three habitats, TLM scaled allometrically with respect to SM, with a common slope of 0.71 (95% CI = 0.64 and 0.79, *P* = 0.81), and the normalization constants of *B. platyphylla* in the lava platform differ significantly in kipuka and Shankou lake.

**Table 3:** Summary of regression slopes and confidence intervals for TLAM, TPM, SM vs TM, TLM vs SM, and ILM vs IPM

Index (log *y* − log *x*)	Item	Slope	95% CI	Intercept	*R* ^*2*^	*P*	*n*
TLAM–TM	H1	1.003	0.97, 1.03	−0.12	0.993	<0.001	30
	H2	0.997	0.98, 1.01	−0.08	0.999	<0.001	30
	H3	1.018	0.99, 1.05	−0.12	0.993	<0.001	30
TPM–TM	H1	0.970	0.86, 1.09	−1.27	0.905	<0.001	30
	H2	1.081	0.89, 1.31	−1.26	0.754	<0.001	30
	H3	0.863	0.74, 1.00	−1.33	0.844	<0.001	30
SM–TM	H1	1.075	0.96, 1.20	−0.74	0.918	<0.001	30
	H2	1.079	1.01, 1.16	−0.94	0.968	<0.001	30
	H3	1.132	0.98, 1.31	−0.73	0.861	<0.001	30
TLM–SM	H1	0.927	0.81, 1.07	0.59	0.871	<0.001	30
	H2	0.920	0.85, 1.00	0.81	0.959	<0.001	30
	H3	0.886	0.75, 1.05	0.56	0.804	<0.001	30
ILM–IPM	H1	1.072	0.88, 1.31	1.08	0.733	<0.001	30
	H2	0.804	0.64, 1.01	1.36	0.650	<0.001	30
	H3	1.217	1.02, 1.46	1.04	0.781	<0.001	30
ILM–LI	H1	−0.999	−1.06, −0.93	2.87	0.972	<0.001	30
	H2	−0.994	−1.01, −0.97	2.92	0.998	<0.001	30
	H3	−1.036	−1.08, 0.99	2.95	0.988	<0.001	30

LI for twig biomass allocations of *B. platyphylla* in a heterogeneous habitat, H1: Kipuka, H2: Lava platform, H3: Shankou lake. Statistically significant relationships are tested at the 0.05 level.

**Figure 2:**
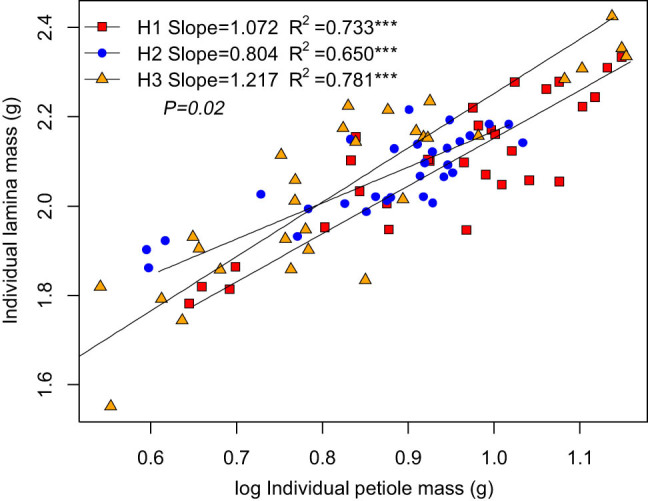
Within leaf biomass allocation relationship of *B. platyphylla*.

The ILM was positively correlated with the IPM (Figure 2, Table 3). Among the three habitats, ILM scaled isometrically with respect to IPM in kipuka and the lava platform (Figure 2), with slopes of 1.072 and 0.804, respectively, but ILM scaled allometrically with respect to IPM in Shankou lake, with a slope of 1.217.

### 3.3 Leaf size-number trade-off of *B. platyphylla* in heterogeneous habitat

**Figure 3:**
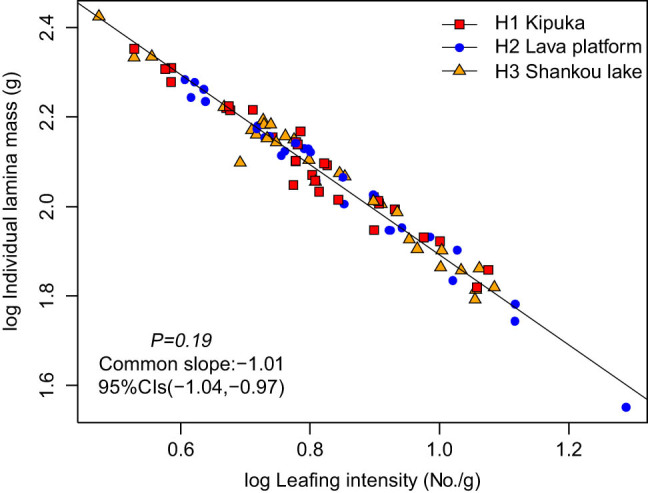
The size-number trade-off of *B. platyphylla* in different habitats.

The ILM was positively correlated with the LI (Figure 3). Among the three habitats, ILM scaled isometrically with respect to LI (Table 3), with a common slope of −1.01 (95% CI = −1.04 and −0.97, *P* = 0.19). The normalization constants did not differ significantly across the three habitats.

## 4 Discussion

Habitat is an important factor affecting the distribution and growth of plants, and the adaptation of plants to habitats is a trade-off process involving internal and external plant functional traits [29]. Different environmental factors, such as soil moisture, nutrient, and light conditions, elicit different plant survival strategies. The LN, TLAM, SM, and TM of *B. platyphylla* were significantly different in the three habitats, suggesting that they are affected by the habitat. They are the greatest in kipuka with good environmental conditions and lowest in the lava platform with relatively poor environmental conditions. This may be because kipuka retains the original dark-brown soil before the eruption, now covered with a layer of black volcanic ash [26]; therefore, soil nutrients are more abundant, and plants produce larger leaves and twigs. Since the volcanic eruption was only 300 years ago, the soil matrix of the lava platform is volcanic stony [26], the soil nitrogen and water content are low, and potassium is rich; in general, plant growth conditions are relatively poor, resulting in smaller leaves and twigs [30,31].

Twig biomass allocation is an important driving factor for plant net carbon acquisition, [32] turnover, and plant life history under different habitats. The leaf is the main site for photosynthesis, where the exchange of material and energy occurs, and the stem has multiple functions of nutrient transport, storage, leaf support, and expansion of growth space. This study found that TLAM and SM were scaled allometrically with respect to TM. TLM scaled allometrically with respect to stem, and the common slope of the TLAM and the TM was lesser than 1. However, the common slope of the SM and the TM was greater than 1. The results showed that the weight increase rate of the stem was higher than the leaf in twigs, probably due to plants preferentially allocate biomass to stem organs to support the leaf and transport nutrients and water in the volcanic habitat. The normalization constants of TLAM and the TM had an upward displacement in the main axis direction in the lava platform. However, the normalization constants of SM and the TM had a downward displacement in the direction of the main axis. The results showed that because of exposed rocks, shallow soil layer, less plant distribution, and, more direct sunlight in the lava platform, *B. platyphylla* needed to allocate a higher proportion of biomass to its leaves for photosynthesis and food production in the lava platform. This study found that the total petiole was isometric with respect to TM with their common slope of 0.96 (95% CI = 0.89 and 1.03, *P* = 0.17); the results showed petiole mass had little effect on biomass allocation in the twig, and petiole mass in the twig was not affected by the habitat.

The leaves have two components: an expanded lamina and a beam-like petiole. The support investment within the leaf is an important part of the support investment of the plant [33]. The lamina produces nutrients, and the petiole is a cantilever structure supporting the lamina and serves in the transportation of nutrients and water. This study found that the slope differs significantly across the three habitats. The results showed that the habitat conditions affected the leaf biomass allocation. The relationship between ILM and IPM was allometric, scaling with slopes significantly >1.0 in the Shankou lake. This suggests that the increase in lamina investment was greater than the lamina support cost. A few studies have found an allometric scaling relationship between the leaves (photosynthetic structures) and petioles (support structures) [25,34]. In general, when the total leaf biomass is not fixed, more biomass is allocated to the leaves, increasing their photosynthetic carbon acquisition capacity; the plant is benefited by increased leaf area and quality. The relationship between ILM and IPM was isometric scaling in kipuka and the lava platform: the higher the lamina mass, the greater the petiole mass. The petiole mass increased to support the increase in the leaf mass [35]. The support investment accelerated with the increased cost of the expanded leaf size, and the increased support cost may be more than offset by the increased carbon absorption due to leaf enlargement.

The plant trade-off relationship between the leaf size and LN is ubiquitous. They are two important factors determining the compactness of the crown that directly affects the canopy structure and the development mode of plants and subsequently affects light interception and carbon acquisition capacity [36]. The changes in leaf sizes are correlated with the availability of water and nutrients in the habitat and other plant functional traits [37]. Sun et al. analyzed the twigs of 123 species datasets compiled in the subtropical mountain forest, the minimum leaf mass and maximum leaf mass versus the LI based on SM (LI), and the scaling exponents ranged from −1.24 to −1.04 [38]. Our study showed a significant negative isometric scaling relationship between ILM and LI in the three habitats. Their common slope was −1.01 (95% CI = −1.04 and −0.97, *P* = 0.19). Previous studies have shown a significant negative isometric scaling between the leaf size and LI for species in different habitats [21,39]. Due to their sessile nature, plants cannot escape risk in the growth process; they take appropriate strategies to adapt to the external environment. The larger leaves are predominantly arranged on the current-year twig in kipuka with the lesser LN, the larger leaves maximizing light interception and photosynthetic carbon acquisition in low light and nutrient-rich habitat [40]; plants will compensate for the increased cost of large-leaf construction and expansion by reducing the number of leaves and minimizing the degree of self-shade. In contrast, *B. platyphylla* had small leaves in the lava platform. Small leaves have a shorter leaf unfolding time than large leaves; they are less resistant to heat and have less surface area for material exchange, which are better adapted to environmental conditions of high light radiation and low nutrients [19], and so, plants need higher LN.

## 5 Conclusion

In this study, LN, TLAM, SM, and TM were significantly different between the three habitats and were greatest in kipuka with abundant soil nutrients. TLAM and SM scaled allometrically with respect to TM, and the normalization constants of the lava platform differ significantly from the kipuka and Shankou lake, which showed that under certain TMs, leaves gain more biomass in the lava platform with barren soil nutrients. However, in within-leaf, ILM scaled isometrically with respect to IPM in kipuka and the lava platform, but ILM scaled allometrically with respect to IPM in Shankou lake. Our results indicated that inhabitats influenced the twig traits and biomass allocation, and within-leaf biomass allocation is a strategy for plants to adapt to volcanic heterogeneous habitats.

## References

[1] Pickup M, Westoby M, Basden A. Dry mass costs of deploying leaf area in relation to leaf size. Funct Ecol. 2005;19(1):88–97.

[2] Zhang H, Song TQ, Wang KL, Yang H, Yue YM, Zeng ZX, et al. Influences of stand characteristics and environmental factors on forest biomass and root-shoot allocation in southwest China. Ecol Eng. 2016;91:7–15.

[3] Warton DI, Weber NC. Common slope tests for bivariate errors-in-variables models. Biom J. 2002;44(2):161–74.

[4] Osada N. Crown development in a pioneer tree, Rhus trichocarpa, in relation to the structure and growth of individual branches. N Phytol. 2006;172(4):667–78. 10.1111/j.1469-8137.2006.01857.x 17096793

[5] Heuret P, Meredieu C, Coudurier T, Courdier F, Barthelemy D. Ontogenetic trends in the morphological features of main stem annual shoots of Pinus pinaster (Pinaceae). Am J Bot. 2006;93(11):1577–87. 10.3732/ajb.93.11.1577 21642103

[6] Barthelemy D, Caraglio Y. Plant architecture: a dynamic, multilevel and comprehensive approach to plant form, structure and ontogeny. Ann Bot. 2007;99(3):375–407. 10.1093/aob/mcl260 PMC2802949 17218346

[7] Westoby M, Wright IJ. The leaf size-twig size spectrum and its relationship to other important spectra of variation among species. Oecologia. 2003;135(4):621–8. 10.1007/s00442-003-1231-6 16228258

[8] Xiang SA, Wu N, Sun SC. Within-twig biomass allocation in subtropical evergreen broad-leaved species along an altitudinal gradient: allometric scaling analysis. Trees-Struct Funct. 2009;23(3):637–47.

[9] Corner EJH. The Durian theory or the origin of the modern tree. Ann Bot. 1949;13(52):367–414.

[10] Mensah S, Kakai RG, Seifert T. Patterns of biomass allocation between foliage and woody structure: the effects of tree size and specific functional traits. Ann For Res. 2016;59(1):49–60.

[11] Wright SJ, Muller-Landau HC, Condit R, Hubbell SP. Gap-dependent recruitment, realized vital rates, and size distributions of tropical trees. Ecology. 2003;84(12):3174–85.

[12] Niinemets U, Portsmuth A, Tobias M. Leaf shape and venation pattern alter the support investments within leaf lamina in temperate species: a neglected source of leaf physiological differentiation? Funct Ecol. 2007;21(1):28–40.

[13] Sun SC, Jin DM, Shi PL. The leaf size-twig size spectrum of temperate woody species along an altitudinal gradient: an invariant allometric scaling relationship. Ann Bot. 2006;97(1):97–107. 10.1093/aob/mcj004 PMC2803375 16254019

[14] Wang M, Jin G, Liu Z. Variation and relationships between twig and leaf traits of species across successional status in temperate forests. Scand J For Res. 2019;34(8):647–55.

[15] Sun J, Wang MT, Cheng L, Lyu M, Sun MK, Li M, et al. Allometry between twig size and leaf size of typical bamboo species along an altitudinal gradient. J Appl Ecol. 2019;30(1):165–72. 10.13287/j.1001-9332.201901.029 30907537

[16] Westoby M, Falster DS, Moles AT, Vesk PA, Wright IJ. Plant ecological strategies: some leading dimensions of variation between species. Annu Rev Ecol Syst. 2002;33:125–59.

[17] Violle C, Navas ML, Vile D, Kazakou E, Fortunel C, Hummel I, et al. Let the concept of trait be functional! Oikos. 2007;116(5):882–92.

[18] Kleiman D, Aarssen LW. The leaf size/number trade-off in trees. J Ecol. 2007;95(2):376–82.

[19] Li T, Deng JM, Wang GX, Cheng DL, Yu ZL. Isometric scaling relationship between leaf number and size within current-year shoots of woody species across contrasting habitats. Pol J Ecol. 2009;57(4):659–67.

[20] Milla R. The leafing intensity premium hypothesis tested across clades, growth forms and altitudes. J Ecol. 2009;97(5):972–83.

[21] Yang DM, Li GY, Sun SC. The generality of leaf size versus number trade-off in temperate woody species. Ann Bot. 2008;102(4):623–9. 10.1093/aob/mcn135 PMC2701782 18682438

[22] Sun J, Wang M, Lyu M, Niklas KJ, Zhong Q, Li M, et al. Stem and leaf growth rates define the leaf size vs number trade-off. Aob Plants. 2019;11:6. 10.1093/aobpla/plz063 PMC6863467 31777650

[23] Feng M, Whitford-Stark JT. The 1719–1921 eruptions of potassium-rich lavas at Wudalianchi, China. J Volcanol Geotherm Res. 1986;30:131–48.

[24] del Moral R, Grishin SY. Volcanic disturbances and ecosystem recovery. In: Wakker LR, editor. Ecosystems of disturbed ground. Amsterdam: Elsevier Science; 1999. Vol. 5. p. 137–60.

[25] Niinemets Ü, Portsmuth A, Tena D, Tobias M, Matesanz S, Valladares F. Do we underestimate the importance of leaf size in plant economics? Disproportional scaling of support costs within the spectrum of leaf physiognomy. Ann Bot. 2007;100(2):283–303. 10.1093/aob/mcm107 PMC2735320 17586597

[26] Zhang SM, Chem LM, Xing RG, Jin KZ. Distribution and features on soil and vegetation of five-linked-great-pool Lake volcano district. Territ Nat Resour Study China. 2005;1:86–8.

[27] Niklas KJ. Plant allometry: the scaling of form and process. Chicago, IL: University of Chicago press; 1994.

[28] Warton DI, Duursma RA, Falster DS, Taskinen S. smatr 3-an R package for estimation and inference about allometric lines. Methods Ecol Evol. 2012;3(2):257–9.

[29] Mooney KA, Halitschke R, Kessler A, Agrawal AA. Evolutionary trade-offs in plants mediate the strength of trophic cascades. Science. 2010;327(5973):1642–4. 10.1126/science.1184814 20339073

[30] Zhao LP, Yang XM, Inoue K. Morphological, chemical, and humus characteristics of volcanic ash soils in Changbaishan and Wudalianchi, Northeast China. Soil Sci Plant Nutr. 1993;39(2):339–50.

[31] Sun CQ, Nemeth K, Zhan T, You HT, Chu GQ, Liu JQ. Tephra evidence for the most recent eruption of Laoheishan volcano, Wudalianchi volcanic field, northeast China. J Volcanol Geotherm Res. 2019;383:103–11.

[32] Korner C. Some often overlooked plant characteristics as determinants of plant-growth – a reconsideration. Funct Ecol. 1991;5(2):162–73.

[33] Niinemets U, Kull K. Leaf weight per area and leaf size of 85 estonian woody species in relation to shade tolerance and light availability. For Ecol Manag. 1994;70(1–3):1–10.

[34] Niinemets U, Portsmuth A, Tobias M. Leaf size modifies support biomass distribution among stems, petioles and mid-ribs in temperate plants. N Phytol. 2006;171(1):91–104. 10.1111/j.1469-8137.2006.01741.x 16771985

[35] Pearcy RW, Yang W. The functional morphology of light capture and carbon gain in the Redwood forest understorey plant Adenocaulon bicolor Hook. Funct Ecol. 1998;12(4):543–52.

[36] Givnish TJ, Vermeij GJ. Sizes and shapes of Liane leaves. Am Nat. 1976;110(975):743–78.

[37] Givnish TJ. Comparative studies of leaf leaf form assessing the relative roles of selective pressures and phylogenetic constraints. N Phytol. 1987;106(1):131–60.

[38] Sun J, Chen XP, Wang MT, Li JL, Zhong QL, Cheng DL. Application of leaf size and leafing intensity scaling across subtropical trees. Ecol Evol. 2020;10(23):13395–402. 10.1002/ece3.6943 PMC7713914 33304546

[39] Xiang SA, Wu N, Sun SC. Testing the generality of the ‘leafing intensity premium’ hypothesis in temperate broad-leaved forests: a survey of variation in leaf size within and between habitats. Evol Ecol. 2010;24(4):685–701.

[40] Poorter H, Pepin S, Rijkers T, de Jong Y, Evans JR, Korner C. Construction costs, chemical composition and payback time of high- and low- irradiance leaves. J Exp Bot. 2006;57(2):355–71. 10.1093/jxb/erj002 16303828

